# The Gut Microbiota in Rheumatoid Arthritis: Evaluation of Microbial Profiles, Disease Activity, Biomarkers, and Treatment Response

**DOI:** 10.31138/mjr.160825.emr

**Published:** 2026-06-12

**Authors:** Danai C. Theiopoulou, Dimitrios Vassilopoulos, Clio P. Mavragani, George Bertsias

**Affiliations:** 1 National and Kapodistrian University of Athens Faculty of Medicine, Athens, Greece;; 2 Joint Rheumatology Program, Clinical Immunology-Rheumatology Unit, 2^nd^ Department of Medicine and Laboratory, National and Kapodistrian University of Athens, School of Medicine, General Hospital of Athens, “Hippokration”, Athens, Greece;; 3 Departments of Physiology and Pathophysiology, School of Medicine, National and Kapodistrian University of Athens, Joint Academic Rheumatology Program, Athens, Greece;; 4 Rheumatology and Clinical Immunology, University of Crete Medical School, Heraklion, Greece

**Keywords:** rheumatoid arthritis, autoimmune diseases, gastrointestinal microbiome, immune system, inflammation, microbiota

## Abstract

**Objectives:**

Rheumatoid arthritis (RA) is a chronic autoimmune disease with complex etiopathogenesis, and variable clinical course. The human gastrointestinal (GI) tract is a dynamic ecosystem in which micro-organisms influence metabolism, immune signalling, and pathogen resistance. Understanding these interactions is key to exploring the microbiota’s role in RA. This review examines how intestinal and oral microbiota differ in RA patients and influence disease activity and treatment response.

**Method:**

A literature search was conducted using PubMed, from inception until January 3, 2025. Studies were selected based on the inclusion of original research involving human subjects diagnosed with RA. Exclusion criteria comprised reviews, case reports, animal studies, and studies with insufficient data. Of the 329 records identified, 72 were included in this review.

**Results:**

Most studies have implicated changes in bacterial composition involving specific phyla in RA-associated dysbiosis. The imbalance is primarily driven by alterations in the phyla *Bacillota*, *Pseudomonadota*, and *Bacteroidota*, with *Bacillota* and *Pseudomonadota* accounting for 70% of increased taxa, and *Bacillota* and *Bacteroidota* for 83% of decreased intestinal taxa. Other findings include reduced α-diversity in seronegative patients and associations between decreased *Prevotella* taxa and higher disease activity, inflammation biomarkers, and autoantibodies. Smaller studies have also reported elevated serum zonulin—a marker of intestinal permeability—in RF-positive patients.

**Conclusion:**

This review highlights key bacterial phyla linked to RA and emphasises the potential of probing gut micro-biota–immune system interactions to improve diagnosis and treatment.

## INTRODUCTION

Rheumatoid arthritis (RA) is a chronic autoimmune disease with a global incidence of around 0.5–1% that affects primarily the joints but can also have systemic involvement.^1,2^ In 2020, an estimated 17.6 million people of all ages were living with RA worldwide, which marks a 121% rise compared to the year 1990.^2^ According to Hootman et al., arthritis is the leading cause of disability, affecting more than 23% of older adults with disabilities.^3^

Growing evidence suggests that interactions between diet, the gut microbiota, and the immune system may contribute to inflammation in a subset of patients with rheumatoid arthritis, indicating that personalised dietary approaches may be beneficial, while other drivers of immune activation should be considered when diet-related mechanisms are absent.^4^ Multiple contributing factors, predominantly genetic and environmental, affect the development of RA. The HLA-DRB1 gene and the PTPN22 tyrosine-phosphatase gene are the strongest genetic influences. The most important environmental contributor is smoking, a major preventable risk factor,^5^ which not only increases susceptibility but is also associated with worse outcomes.^6^ Other possible factors include air pollution and inhaled exposure to substances other than cigarette smoke, hormone fluctuations in women, diet, alcohol consumption, vita-min D levels, certain infectious diseases, geographical location, and socioeconomic status.^7–9^

**Figure 1.**
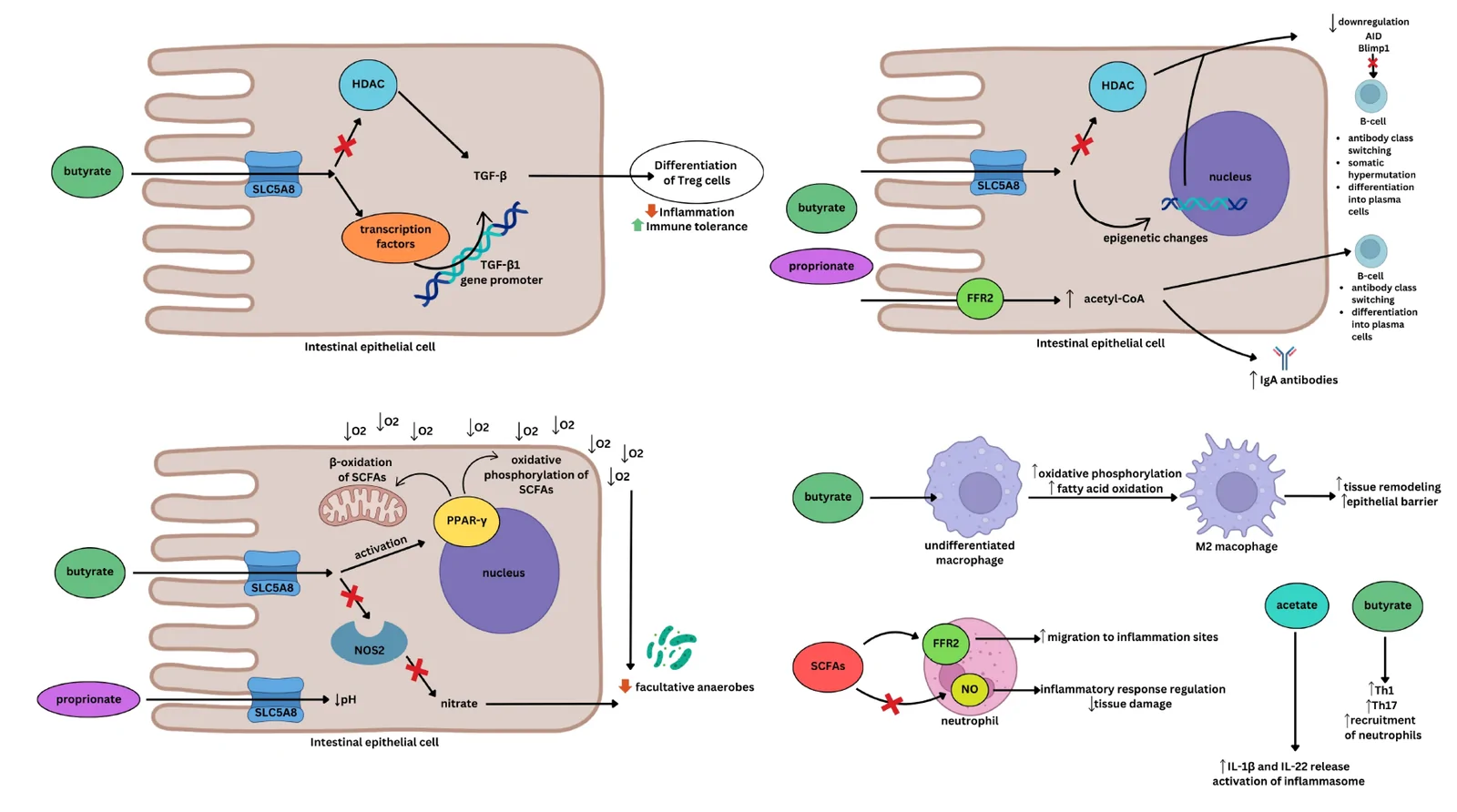
SCFA metabolism and immune modulation.

The effect of the gut microbiota on immune system homeostasis has been hypothesised as early as 1965 in a study by Colldahl et al.^10^ In recent times, several studies have revealed various pathophysiologic mechanisms explaining this potentially significant link and implicate specific microorganisms. The gut microbiota supports mucosal barrier integrity, shapes the intestinal epithelium, synthesises essential nutrients, protects against pathogens, and regulates immunity.^11,12^ Bacteria in the colon have enzymes that can ferment complex carbohydrates and generate metabolites, such as short-chain fatty acids (SCFAs), primarily butyrate, propionate, and acetate.^13^ These SCFAs influence immune homeostasis by modulating intestinal epithelial cell metabolism, promoting anti-inflammatory responses (e.g., Treg and M2 macrophage differentiation), limiting pathogen growth, and regulating B-cell and neutrophil functions through receptor signalling and epigenetic changes.^14^ Their effects depend on the surrounding microenvironment and the presence of pro- or anti-inflammatory cytokines, as seen in **Figure 1**.

Proposed mechanisms linking the gut microbiota to RA autoantibody production include protein citrullination by PAD enzymes, which can lead to the production of anti-citrullinated protein antibodies (ACPAs) that attack joint tissue.^15,16^ Molecular mimicry is another presumed pathway, where microorganisms activate immune cells that later mistakenly target the joints.^17^ In addition, gut dysbiosis has been associated with auto-immune responses via Th17 cells, as shown in mouse models where Segmented Filamentous Bacteria (SFB) induced arthritis.^18,19^ Other bacteria, such as *Collinsella aerofaciens* and *Prevotella copri*, have been associated with increased intestinal permeability and promoting the production of pro-inflammatory cytokines, which then enter the bloodstream and affect distant sites.^20,21^ Studies have shown a significant similarity between the intestinal and oral microbiota in RA patients, suggesting that they share common bacterial species and metabolic function. For instance, Zhang et al. reported that *Lactobacillus salivarius* was enriched at both sites in RA patients compared to healthy controls and was correlated with higher IgG levels and increased disease severity.^22^ They also suggested that the oral and gut microbiota in RA patients share parallel functional alterations, including reduced secretion systems, polyamine transport/synthesis, and ubiquinone production, alongside increased menaquinone synthesis and a shift in respiratory chain activity (Complex I ↑; Complex III & IV ↓), indicating a common shift toward altered anaerobic energy metabolism.^22^ A similar pattern was observed in the viral microbiome, as Guo et al. reported that 77.9% of viral operational taxonomic units (OTUs) were shared between the oral and gut viromes.^23^ A recent analysis of 503 Scopus-indexed publications revealed a consistent increase in global research output on rheumatic diseases and the gut microbiota from 2002 to 2022, highlighting substantial international contributions and growing scientific interest in this field.^24^

The intricate connection between the gut microbiota and the immune system presents valuable opportunities for improving both diagnostic and therapeutic strategies in RA. Here, we critically examine current evidence on differences in the intestinal and oral microbiota between individuals with RA and healthy controls. We also explore how these microbial alterations relate to disease activity, key laboratory findings, and treatment response, highlighting well-established insights and pointing to gaps that warrant further investigation.

## MATERIALS AND METHODS

### Objectives

This narrative literature review aims to address several key questions regarding the role of the gut microbiota in the development and expression of RA. Specifically, it explores whether the gut microbial profile of RA patients differs from that of healthy individuals and examines its potential influence on disease activity. Possible associations between the microbiota and levels of rheumatoid factor (RF) or anti-citrullinated protein antibodies (ACPA) are also examined, as well as with inflammation biomarkers and specific cytokines. Additionally, this review investigates the potential bi-directional relationship between the gut microbiota and RA treatments. Finally, common limitations are presented after a quality appraisal of the literature.

### Information sources and search strategy

Systematic searches were performed using PubMed/MEDLINE, from inception until the 3rd of January 2025. Other databases could not be accessed due to institutional limitations at the current time. The following search strategies were used to identify relevant literature, as seen in **Table 1**.

**Table 1.** Search terms and strategies.

**Search strategy 1:** (microbiota[Title]) AND (rheumatoid arthritis[Title])
**Search strategy 2:** (microbiome[Title]) AND (rheumatoid arthritis[Title])
**Search strategy 3:** (((gut[Title]) AND (rheumatoid arthritis[Title])) NOT (microbiome)) NOT (microbiota)
**Search strategy 4:** (dysbiosis[Title]) AND (rheumatoid arthritis[Title])
**Search strategy 5:** (((intestinal[Title]) AND (rheumatoid arthritis[Title])) NOT (microbiome[Title])) NOT (microbiota[Title])
**Search strategy 6:** ((rheumatoid arthritis[Title]) AND (flora[Title])) NOT (intestinal[Title])

### Selection and data collection process

To determine whether a study met the inclusion criteria of the review, all articles were manually screened and sorted accordingly. Data was manually extracted and input into a categorised table. All records were screened and reviewed once, by a single reviewer working independently. No automation tools were used in either process.

### Inclusion and exclusion criteria

The inclusion and exclusion criteria can be seen in **Table 2.** Although most included studies included healthy controls, those comparing RA patients to a different disease group were not omitted, though these findings were analysed separately. The criteria used to diagnose RA were not evaluated, and the accuracy of the diagnosis was left to the authors’ discretion. Research published at any time was included; there were no limitations applied on the year of publication. Likewise, there were no geographical, ethnic, age, gender, or other limiting criteria placed.

**Table 2.** Inclusion and exclusion criteria.

**Inclusion Criteria**	**Exclusion Criteria**
Original research studies Human subjects Results relevant to the topic	Non-original findings Case reports Animal studies Results not relevant to the topic Insufficient data Duplicates

### Data items

Data were explored for any reported differences in the gut microbial profile of RA patients compared to control groups, disease activity, RF, ACPA, CRP, ESR, other biomarkers, and response to treatment. To provide a more comprehensive review, data on the oral microbiota were also included, as the oral cavity is part of the gastrointestinal tract. To study differences in microbial profiles, information on α- and β-diversity as well as specific microorganisms was extracted. All results relevant to each outcome domain were included, regardless of measures, time points, or analytic approach. No additional selection methods were applied.

Data were sought for other variables as well, more specifically for: year and journal of publication, type of study, study population, aim of study, limitations, samples used, methods used, relevant pathophysiologic mechanisms mentioned, and overall conclusions.

The search strategy was informed by recommendations on comprehensive literature searches endorsed by the Mediterranean Journal of Rheumatology; however, given the narrative nature of this review, a focused search of PubMed/MEDLINE was undertaken.

## RESULTS

### Study selection

**Figure 2.**
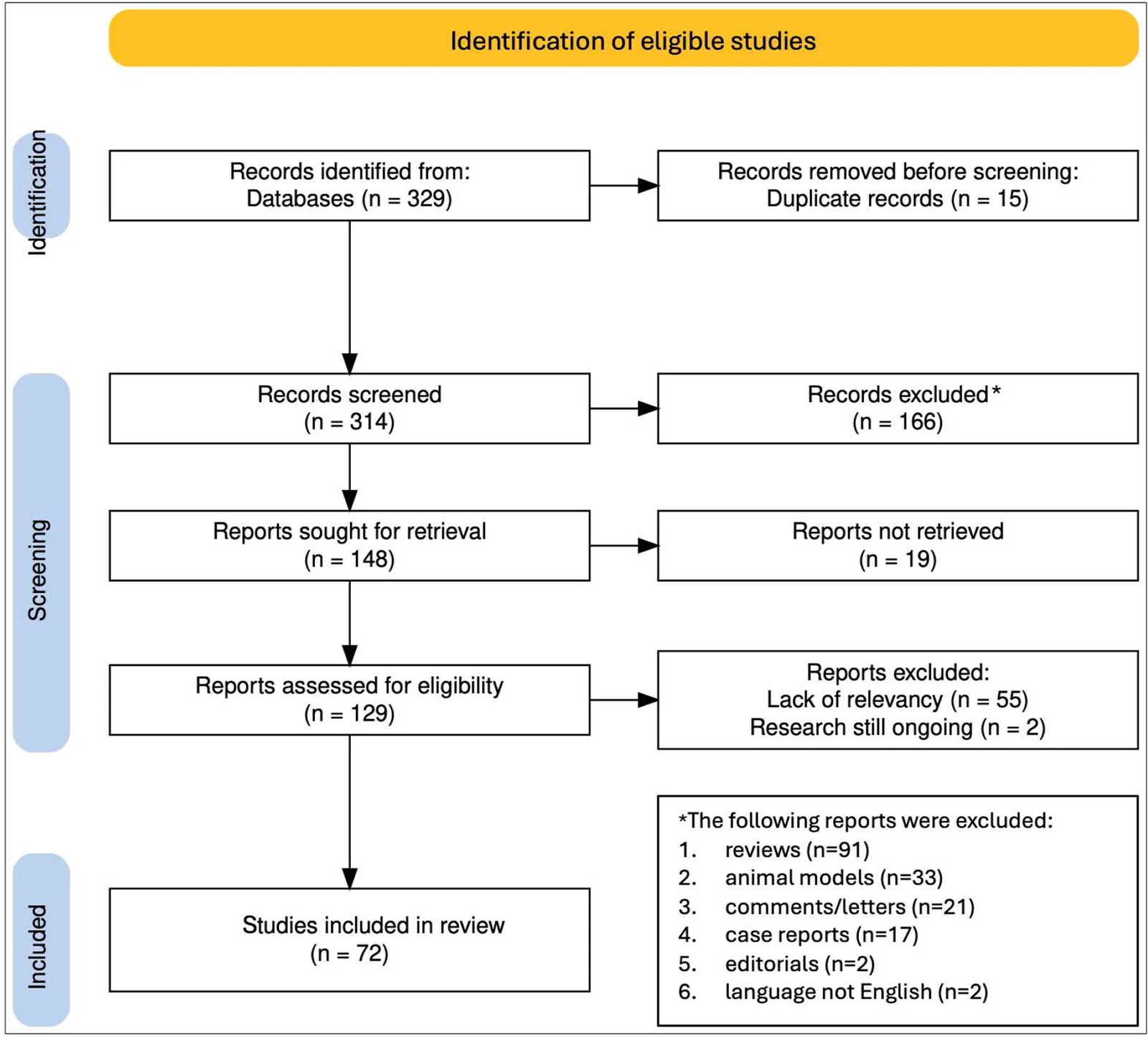
Results of the search and selection process.

The results of the search and selection process are shown in greater detail in **Figure 2**.

### Study characteristics

Included studies and their characteristics can be found in **Annex 1**.

### Differences in gut microbial profiles between RA patients and healthy controls

Of the 72 included studies, 46 compared RA patients’ gut or oral microbial profile to that of healthy individuals. Many studies reported gut dysbiosis in RA, the presence of abnormal flora or intestinal bacterial overgrowth, supported by the differences in diversity and composition of microorganisms. For instance, Nurgaziyev et al.^25^ reported significant differences in α-diversity as shown by the Chao 1 index (p = 0.019) and Faith’s PD (p = 0.006) of the gut microbiota. Some studies reported that RA patients showed decreased α-diversity, namely Chen et al.,^21^ Chiang et al.,^26^ Sun et al.,^27^ Tong et al.,^28^ Kim et al.,^29^ Luo et al.^31–36^ However, others found no significant change in α-diversity when compared to healthy control groups or other disease groups such as osteoarthritis.^37^ One study had conflicting results when using different methods of measuring α-diversity.^38^ However, all studies examining β-diversity reported a statistically significant difference between RA patients and healthy controls.^25,34,35,39–41^

**Figure 3.**
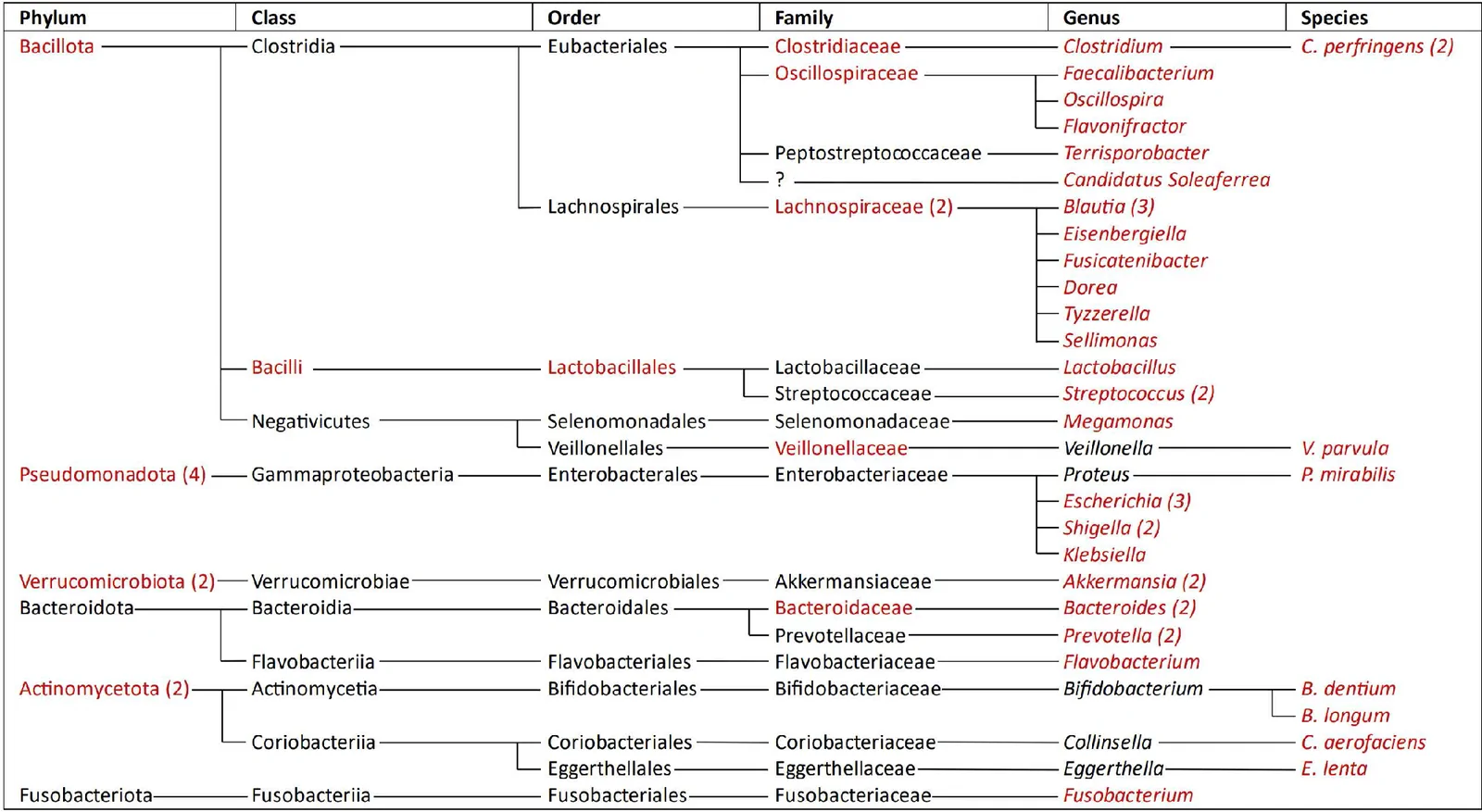
Intestinal taxa found to be increased in RA patients compared to HCs.

**Figure 4.**
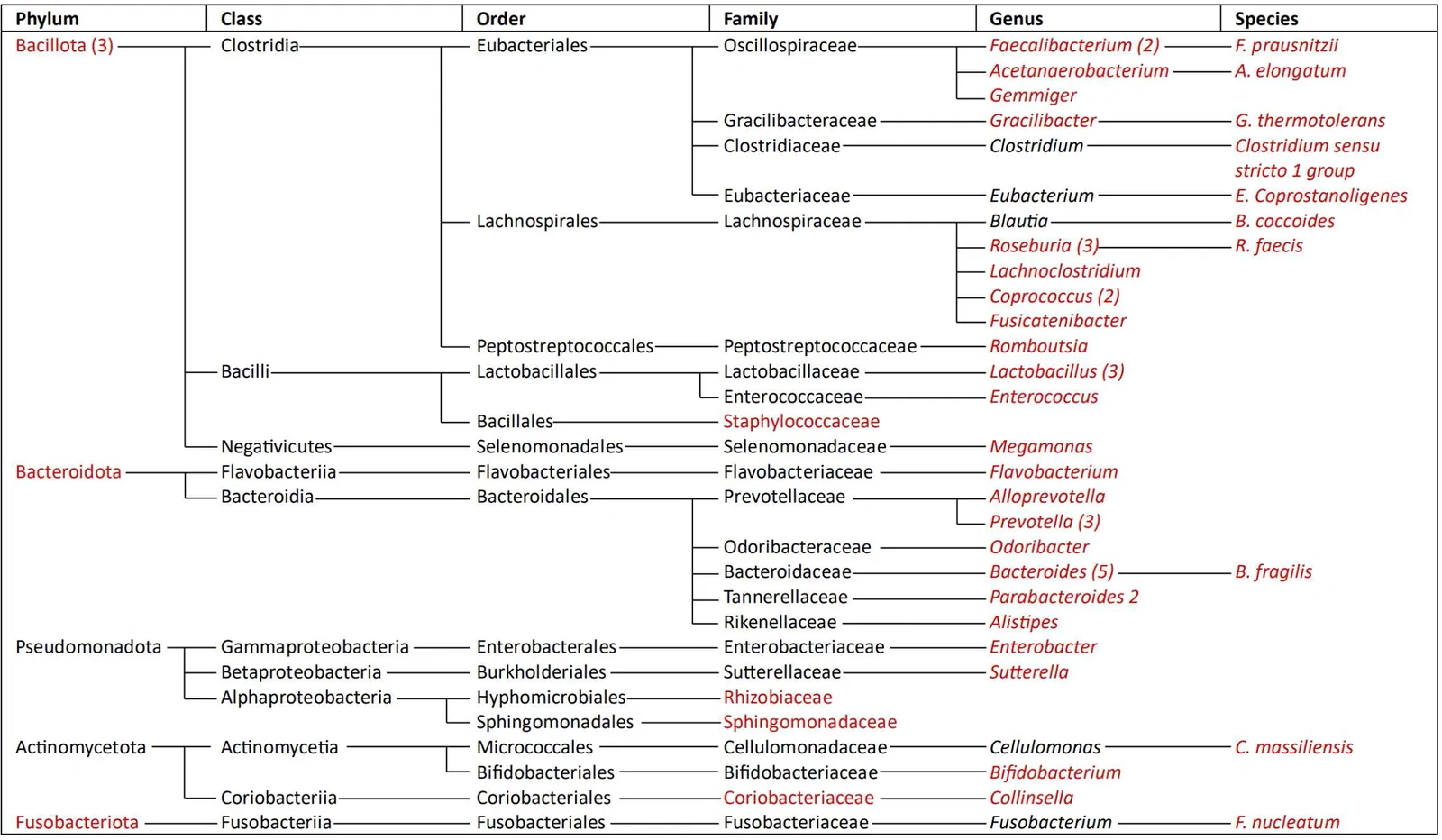
Intestinal taxa found to be decreased in RA patients compared to HCs.

**Figure 5.**
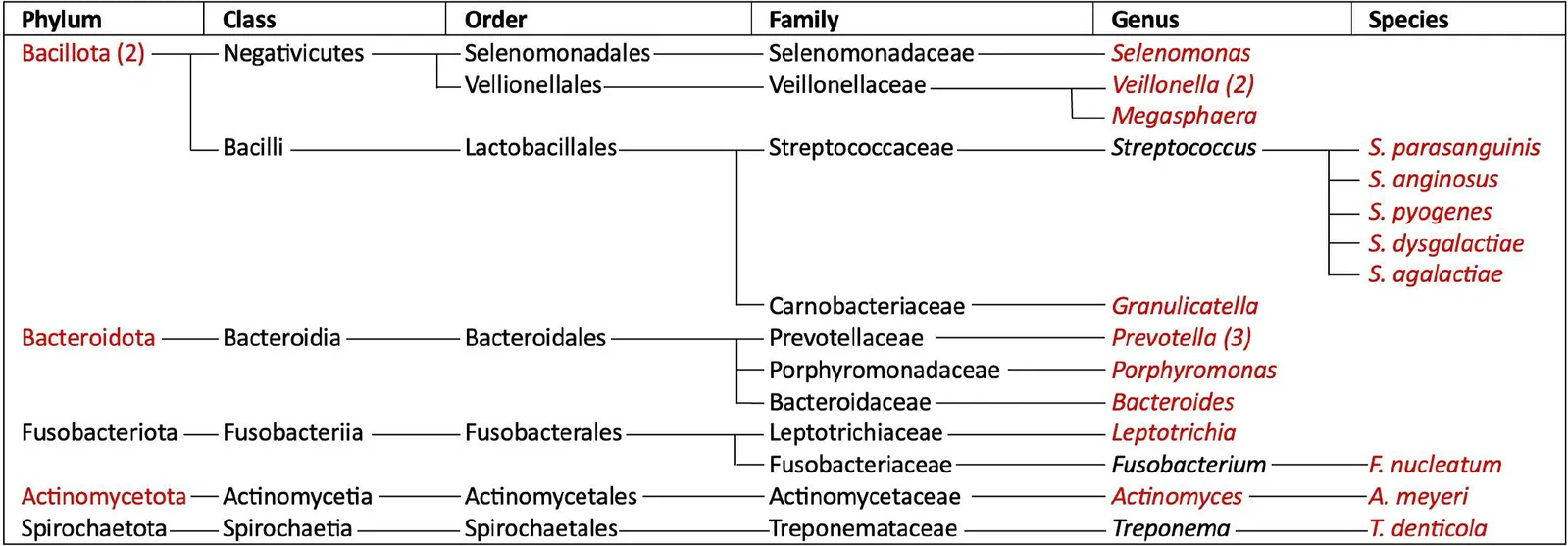
**(above).** Oral taxa found to be increased in RA patients compared to HCs.

**Figure 6.**
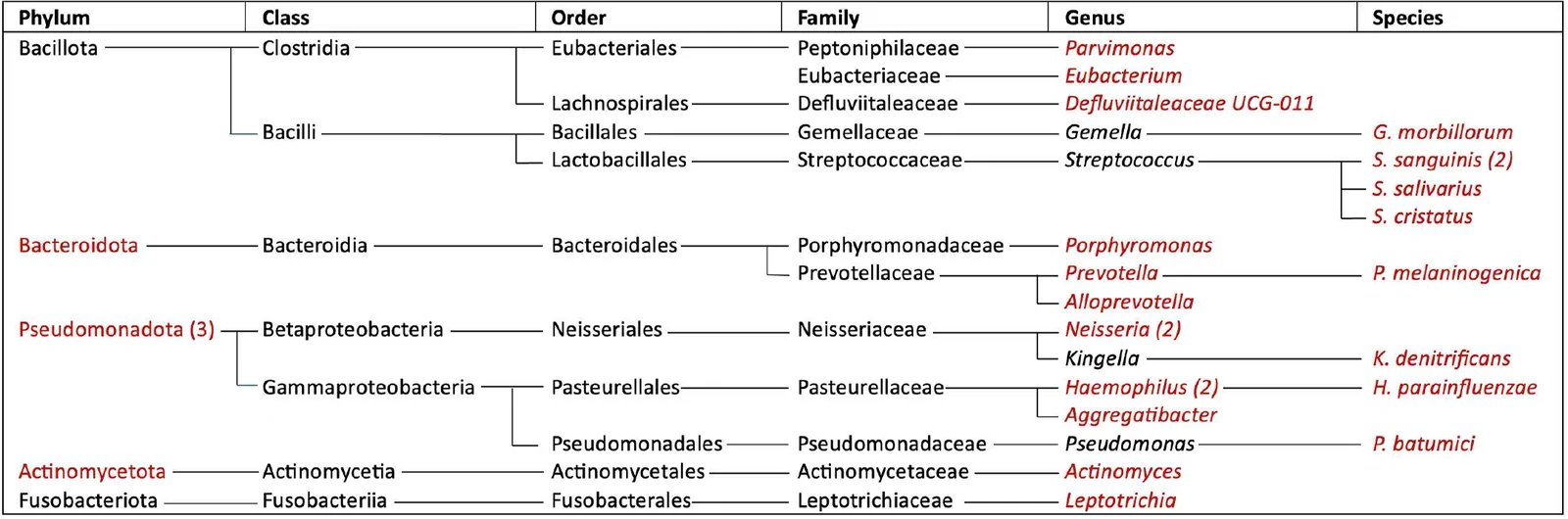
**(below).** Oral taxa found to be decreased in RA patients compared to HCs.

When exploring differences in microbial composition, results were diverse. To better visualise the relationships between the implicated taxa, they were organised based on their phylogenetic traits, as illustrated in **Figure 3** (Intestinal taxa found to be increased in RA patients compared to HCs), **Figure 4** (Intestinal taxa found to be decreased in RA patients compared to HCs), **Figure 5** (Oral taxa found to be increased in RA patients compared to HCs), and **Figure 6** (Oral taxa found to be decreased in RA patients compared to HCs). The taxa shown in **Figures 3–6** represent a qualitative synthesis across the included studies. Microorganisms were identified by manually reviewing each publication and recording whether they were reported as increased or decreased in rheumatoid arthritis or in relation to clinical variables. The figures summarise how frequently specific taxa were reported across studies and do not represent pooled quantitative effect sizes or meta-analytic results.

**Figure 7.**
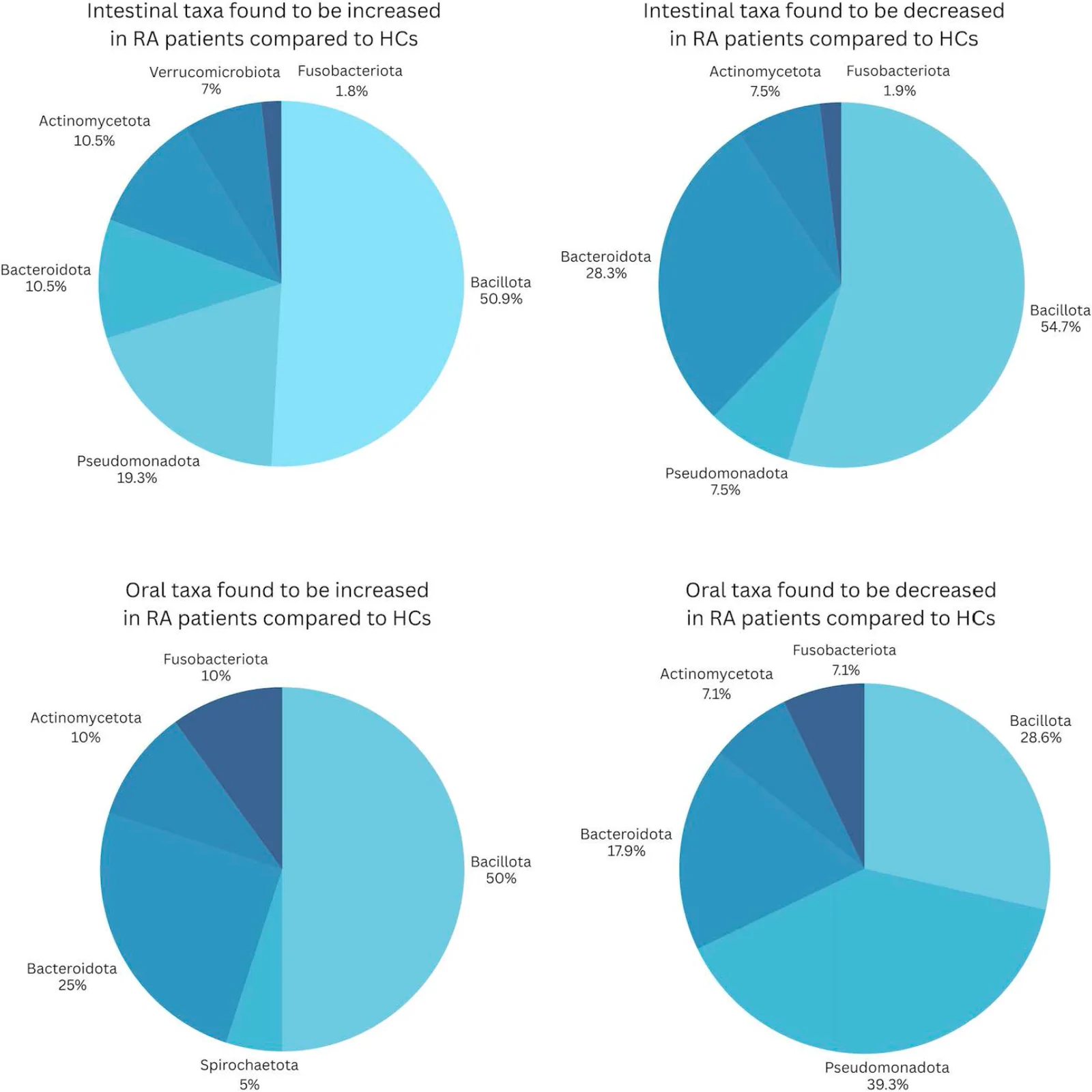
Percentages of bacterial phyla that are dysregulated in the intestine or oral cavity of rheumatoid arthritis patients compared to healthy controls.

Across all figures, *Bacillota* emerges as the most consistently implicated phylum, showing significant associations with both increases and decreases in taxa in RA patients, depending on the site (gut or oral cavity). *Pseudomonadota* also features prominently, particularly in taxa that are decreased, while *Bacteroidota* plays a key role in both increases and decreases. As seen in **Figure 7,** (percentages of bacterial phyla that are dys-regulated in the intestine or oral cavity of rheumatoid arthritis patients compared to healthy controls), *Bacillota* and *Pseudomonadota* combined comprise 70% of the increased intestinal taxa, while *Bacillota* and *Bacteroidota* combined account for 83% of the decreased intestinal taxa. These patterns suggest that microbial imbalances in these phyla are commonly observed in RA-associated dysbiosis, warranting further investigation into their possible relevance for disease mechanisms and therapeutic strategies.

One study reported statistically significant differences in the fungal composition of the gut in RA patients compared to HCs. According to Sun et al.,^42^ the following fungal genera were increased in RA patients: *Wallemia, Phanerochaete, Suhomyces, Tolypocladium, Trebouxia, Tremella, Scolecostigmina*, and *Sarocladium*. Genera that were decreased compared to HCs included: *Ganoderma, Auricularia, Pholiota, Aureobasidium, Pleurotus, Trichosporon, Podosphaera, Dipodascus*, and *Alternaria*. These findings indicated that fungal dysbiosis was present in RA patients, even though many of the identified genera were not highly abundant.

### The gut microbiota in relation to RA disease activity

**Table 3** summarises key findings from studies examining the relationship between microbiota and RA disease activity. Most studies reported associations between specific microbial taxa and disease activity scores, inflammation, or treatment response, particularly highlighting changes in gut and oral microbial composition. However, a few studies found no significant correlation, suggesting that while the evidence supports a link, the interaction is likely complex and influenced by multiple factors.

**Table 3.** Associations between microbiota and RA disease activity.

**Study**	**Finding**	**Organism or Parameter Involved**
Henriksson et al.^42^	Higher disease activity in RA patients with bacterial overgrowth	General overgrowth (small intestinal)
Chen et al.^21^	Reduced diversity linked to longer disease duration	↓ Diversity, ↑ Eggerthella, ↓ Faecalibacterium
Picchianti et al.^30^	DAS28 positively associated with some taxa, negatively with others	↑ Euryarchaeota, Gammaproteobacteria, Pasteurellales, Anaerobranca; ↓ Erysipelotrichia, Coriobacteriaceae, Collinsella
Chianq et al.^25^	↑ Collinsella in active RA	Collinsella
Tong et at.^27^	Positive correlation with DAS28	Ruminococcaceae UCG-014
Wang et al.^43^	Positive association with DAS28	Bifidobacterium, Lachnospira, Blautia, Muribaculaceae
Yun et al.^44^	Negative correlation with DAS28	Sutterella
Andreasson et al.^45^	Improvement in DAS28 linked to ↓, Prevotella copri	Prevotella copri
Rooney et al,^46^	RA progression in at-risk individuals linked to shifts in Prevotellaceae strains	Prevotellaceae
Haupl et al.^47^	Microbiota reduction reduced disease activity	General microbiota reduction
Peltonen et al.^48 49^	Fasting + vegan/lactovegetarian diets improved symptoms; linked to distinct gut microbiota	Diet-modified microbiota
Guan et al.^50^	↑ Serum zonulin (gut permeability) associated with longer RA duration	Zonulin (marker of permeability)
Cheng et al..^51^	Dysbiosis and ↑ permeability promoted inflammation and bone damage	Dysbiosis, bacterial translocation
He et al.^32^	↓ Butyrate (anti-inflammatory SCFA) linked to ↑ disease activity and joint deformities	↓ Fecal/serum butyrate
Beyer et al.^62^	↓ Subgingival microbiome diversity in active RA	Oral microbiota diversity
Cheah et al.^40^	Subgingival dysbiosis linked to higher DAS 28	Campylobacter
Oliveira et al.^53^	Similar to Cheah et al.; dysbiosis present even without periodontitis	Oral microbiota
Zaragoza-Garcia et al.^54^	Lactobacillus/Porphyromonas gingivalis ratio shifts with disease activity	Oral bacteria ratio
Li et al.^35^	Tonsillar microbiota influenced by disease activity	Tonsillar microbiota
Olhagen et.al.^55^	No association with joint damage	Clostridium perfringens
Eerola et al.^56^	Microbial differences between RA and controls, but not with disease duration	General microbiota
Mikuls et al.^36^; Koh et al.^33^; Rooney et al.^57^	No significant link between microbiota and disease activity	Microbial diversity

### The gut microbiota in relation to RA autoantibodies

We found a number of studies that reported a significant correlation between gut and oral microbiota and the presence of RF and/or ACPAs in RA. Scher et al.^43^ found an association between *Aeroglobus* and ACPAs (P <0.05), while Yun et al.^44^ identified strong positive correlations between RF and *Megamonas*, *Dialister*, and *SMB53*. Oliveira et al.^45^ reported a link between RF and *Lachnospiraceae.NK4A136 (rho = 0.422; p = 0.028)*. Picchianti et al.^31^ found both RF and ACPAs to be positively associated with *Roseburia* and negatively with *Bacilli*, *Lactobacillales*, and *Streptococcus vestibularis*. ACPA-positivity was also linked to *Streptococcaceae*, *Erysipelotrichaceae*, *Streptococcus*, *Bacteroides xylanisolvens*, and *Lachnospira pectinoschiza*.

Chiang et al.^26^ observed higher levels of *Blautia* and *Collinsella* in RF-positive patients, and increased *Blautia*, *Akkermansia*, and *Clostridiales* in ACPA-positive ones. Lower α-diversity among seronegative patients was reported by Chiang,^26^ Chen,^21^ Rooney,^46^ and Li,^36^ while Rooney et al.^47^ also found β-diversity differences between ACPA-positive patients and healthy controls. Numerous other taxa showed positive correlations with RF or ACPA, including *Fusobacteria*, *Saccharibacteria*, *Ruminococcaceae*, *Porphyromonadaceae*, *Dorea*, *Ruminococcus*, *Prevotella*, *Actinomyces*, and *Odoribacter*.^25,27,28,47–49^ Negative correlations were reported with *Bacteroidaceae*, *Alloprevotella*, *Faecalibacterium*, *Colidextribacter*, and *Enterococcus faecalis*. ^25, 32,47,50^

Indirect evidence also exists: Heidt et al.^51^ found elevated serum zonulin in RF-positive patients, Eerola et al.^52^ noted shared fatty acid profiles between RF-positive and erosive arthritis groups, and He et al.^33^ observed shifts in butyrate-metabolising bacteria between ACPA-positive and ACPA-negative patients.

Some studies, including those by Olhagen,^53^ Koh,^34^ Beyer,^54^ and Rooney,^47^ reported no association between microbial composition and seropositivity.

In conclusion, while multiple studies support an association between specific gut microbiota profiles and RF or ACPA status, variability in study design and populations limits the strength of the conclusions that can be drawn.

### The gut microbiota in relation to inflammation biomarkers

Several studies have demonstrated associations between gut microbiota and inflammation biomarkers such as erythrocyte sedimentation rate (ESR) and C-reactive protein (CRP) in RA. Olhagen et al.^53^ reported a positive correlation between *Clostridium perfringens* and ESR. Picchianti et al.^31^ found direct correlations between CRP and *Parabacteroides distasonis*, and between ESR and *Enterobacteriales*, *Roseburia faecis*, and *Streptococcus parasanguinis*. Similarly, Sun et al.^27^ showed that *Alloprevotella* and *Parabacteroides* correlated positively with ESR, while *Prevotella-2* and *Alloprevotella* correlated with CRP. Tong et al.^28^ identified associations of *Ruminococcaceae_UCG-014*, *Lactobacillus*, and *norank_f__Saccharimonadaceae* with ESR, CRP, or both, whereas *Oribacterium* was negatively correlated with CRP. Oliveira et al.^45^ reported correlations between CRP and *Leptotrichia* in oral microbiota, and *Butyricimonas*, *Eubacterium eligens*, and unclassified *Lachnospiraceae* in the gut. Only Beyer et al.^54^ found no significant link between CRP or ESR and microbial diversity or composition.

Beyond general inflammatory markers, several studies reported relationships between the microbiota and specific inflammatory cytokines or immune cells. Zaragoza-García et al.^55^ linked shifts in the *Lactobacillus/Porphyromonas gingivalis* ratio with increased serum IL-17A in RA patients (r = 0.778, p = 0.030). Chiang et al.^26^ found increased *Gammaproteobacteria* and decreased *Bifidobacterium* in patients with elevated TNF-α or IL-17A, and higher *Euryarchaeota* in those with increased IL-6 or IL-17A. Li et al^56^ observed positive correlations between *Pelagibacterium*, *Oxalobacter*, *Clostridium XIVb*, and *Clostridium XVIII* with cytokines, while *Clostridium XIVa*, *Blautia*, and *Ruminococcus2* were more abundant in patients with lower T-cell, B-cell, CD4+ T-cell, and Treg counts. Wang et al.^57^ reported *Megamonas*, *Monoglobus*, and *Prevotella* positively associated with CD4+ T-cell counts and cytokines, including IL-10, IL-2, IL-4, TNF-α, and IFN-γ. Additionally, *Muribaculaceae* and *Odoribacter* correlated with IL-17, TNF-α, and IFN-γ, while *Prevotella* was linked to IL-4. Qiao et al.^58^ classified RA patients by gut enterotypes: E1 (Prevotella dominant) and E2 (Bacteroides dominant), with the latter showing higher CD8+ T-cell counts (P < 0.05). Nurgaziyev et al.^25^ identified diverse correlations between taxa and cytokines or immunoglobulins: *Sellimonas* was positively associated with pro-inflammatory cytokines (TNF-α, IL-17A, IFN-γ) but negatively with immunoglobulins (IgM, IgG subclasses); *Clostridium sensu stricto 1* positively linked to chemokines and cytokines but negatively with IgG2; *Ruminococcus* positively related to MCSF, TNF-β, IL-5 and negatively with various immunoglobulins; *Dorea* had positive associations with MCSF and IL-5 but negative with IgM and IgG3; *Tyzzerella* was positively correlated with MCSF and negatively with IgA; *Bacteroides* positively associated with IL-10 and MCSF; *Lachnospiraceae NK4A136 group* and *Eubacterium siraeum group* were negatively associated with PDGF AB/BB; *Collinsella* correlated positively with IgG2; and *Staphylococcus* linked positively to IL-13.

Eriksson et al.^59^ further found that the pro-inflammatory mediator TWEAK/TNFSF12 positively correlated with *Sphingobium*, *Aquabacterium*, *Novosphingobium*, and *Acidovorax delafieldii*. Pentraxin-3 was positively linked to *Aquabacterium* and *Acidovorax delafieldii*, and IL-19 similarly correlated with *Acidovorax delafieldii*. Overall, associations between gut microbiota composition and inflammatory biomarkers in rheumatoid arthritis vary considerably across studies, reflecting differences in disease activity, treatment status, and analytical approaches, and should therefore be interpreted with caution. Specific microbial taxa have been associated with differences in immune responses, including variations in cytokine levels and immune cell populations, highlighting the gut microbiota as a promising area for biomarker development and future therapeutic investigation in RA. Understanding these mechanisms will enhance insight into RA pathophysiology and guide future research on microbiota-driven inflammation modulation.

### The bi-directional link between the gut microbiota and RA treatments

Here, we also explored the interplay between the gut microbiota and RA treatments. Several studies examined sulfasalazine (SSZ): Bradley et al.^60^ found no significant association between bacterial composition and treatment response, despite decreases in Clostridium perfringens among poor responders. Guan et al.^61^ reported reduced richness and evenness, with increased *Lactobacillus sakei*, *Christensenellaceae* R7, and *Coprococcus*, and decreased *Clostridium sensu stricto 1*, *Roseburia*, and *Turicibacter*. Neumann et al.^62^ observed reduced E. coli and C. perfringens in SSZ-treated patients compared to those on D-penicillamine.

MTX and hydroxychloroquine (HCQ) were linked to higher microbial diversity, with HCQ users showing more *Faecalibacterium* (Chen et al.^21^). Picchianti et al.^31^ reported stable α-diversity in treatment-naïve and treated patients but found distinct shifts in those on etanercept (ETN), including increased *Cyanobacteria* and decreased *Deltaproteobacteria* and *Clostridiaceae*. MTX also reduced *Enterobacteriales* abundance.

In a randomised trial, Mei et al.^32^ showed that patients on a herbal formula (HQT) plus MTX had more pronounced microbial and metabolic shifts than those on leflunomide plus MTX. HQT treatment altered 11 species and 9 pathways, especially purine degradation and amino acid biosynthesis.

Other studies linked microbial signatures to treatment exposure or response. Zaragoza-García et al.^55^ found inverse correlations between total MTX/prednisone dose and *Lactobacillus*, and a positive association between prednisone and *Bacteroides fragilis*. Jiang et al.^63^ noted reductions in *Ruminococcus* and *Faecalibacterium* with csDMARDs, while Andréasson et al.^64^ reported greater microbiota normalisation (Dysbiosis Index Score) with TNF inhibitors than MTX. These findings indicate that biologic therapy may exert a stronger normalising effect on gut microbiota composition than conventional DMARDs.

Oral microbiota was also affected. Beyer et al.^54^ found prednisolone use associated with facultative anaerobes, while non-users had more strict anaerobes like *Fretibacterium* and *Treponema*. Microbial profiles differed by DMARD exposure.

Some studies investigated predictive microbial markers for treatment response. Artacho et al. demonstrated that MTX non-responders had lower microbial diversity at baseline and identified 38 orthologous genes enriched in non-responders, suggesting that pretreatment microbiome composition may influence MTX efficacy.^65^ Han et al. used a random forest algorithm to identify MTX-metabolising genes predictive of response. They found that the metabolic capacity of the gut microbiota, inferred from metagenomic profiles, was associated with rheumatoid arthritis and could help identify microbial gene patterns predictive of treatment response. Specifically, genes involved in methotrexate metabolism and folate pathways were enriched in patients who showed a better therapeutic response.^66^ Guan et al.61 suggested higher *Fusicatenibacter* and *Subdoligranulum* abundance in csDMARD responders. Häupl et al.^67^ linked *Abiotrophia* to MTX non-response, while Qiao et al.^58^ found better MTX response in *Prevotella*-dominant vs. Bacteroides-dominant enterotypes. This enterotype-specific difference suggests that baseline gut microbiota structure may be associated with differences in therapeutic response to MTX and could potentially inform patient stratification.

Finally, Danckert et al.^49^ showed that DMARD monotherapy had minimal impact on microbiota, while MTX combinations altered β-diversity. Eighteen taxa differed by response status after six weeks, with decreased *Prevotella* and *Streptococcus* species suggesting microbiota normalisation in responders. Importantly, Danckert et al. showed that early clinical response to MTX-based therapy was accompanied by a shift toward a micro-biota profile resembling healthy controls, supporting the concept of microbiota normalisation in responders. Similarly, Marazzato et al.^68^ found MTX treatment was associated with shifts in microbiota composition toward profiles resembling those of healthy controls.

### Quality appraisal of the literature

The studies included in this review have several notable strengths, including the integration of clinical parameters such as disease activity, biomarkers, and treatment response, as well as the use of advanced microbiome analysis techniques. Many employed well-designed methodologies, such as case-control or longitudinal approaches with appropriate comparison groups, which strengthened the reliability of the observed associations between microbiota composition and RA outcomes. Additionally, some studies explored mechanistic links between specific microbial taxa and immune pathways, emphasising the biological relevance of their findings.

However, the review also identified important limitations, such as the lack of ethnic diversity and small sample size which increase the risk of bias. Methodologically, many studies relied on 16S rRNA sequencing or DIS scores, which provide limited views of bacterial strains. Metagenomic sequencing offered fuller insights. Most studies focused on bacteria only, neglecting other microbiota components like viruses, fungi, and archaea, limiting understanding of microbial complexity. Additionally, follow-up periods were short, making the assessment of long-term effects on RA progression impossible. Patient selection was another challenge. Most studies recruited long-standing RA cases, excluding early-stage or new-onset RA, and often lacked control groups. Participants were predominantly female, which limits generalisability. Some studies did not account for patients’ treatments, introducing confounding factors. Though some studies suggest microbiota influence treatment response, mechanisms remain unclear. These limitations highlight the need for larger, more diverse cohorts, more advanced sequencing techniques, and longer follow-up periods to better understand the microbiota’s role in RA pathogenesis.

## LIMITATIONS

This narrative review has several limitations. The literature search was limited to PubMed/MEDLINE, which may have resulted in the omission of relevant studies indexed in other databases. Study selection and data extraction were performed by a single reviewer, potentially introducing selection bias. Furthermore, while the review addresses broad questions regarding microbiota profiles and disease activity in rheumatoid arthritis, the narrative design does not allow for the quantitative synthesis achievable in systematic reviews; therefore, conclusions should be interpreted with appropriate caution. Quantitative statistical measures (e.g., p-values, correlation coefficients, or effect sizes) were reported when available in the original studies; however, inconsistent statistical reporting and substantial methodological heterogeneity across studies prevented uniform quantitative comparison.

## CONCLUSION

This review adds to existing literature by integrating evidence from human studies that link gut and oral microbiota alterations with rheumatoid arthritis disease activity, serological markers, inflammatory biomarkers, and treatment response. By synthesising findings across these domains, rather than addressing them in isolation, this work provides a more comprehensive perspective on microbiota–disease interactions and highlights their potential relevance for patient stratification and future precision medicine approaches. The findings underscore the importance of the microbiota in chronic diseases and contribute to a deeper understanding of its role in RA development and expression. This review lays the foundation for future research by identifying the most commonly implicated phyla and drawing common conclusions across the literature. Data from this review suggest that the microbiota may be relevant to disease stratification and treatment response in RA. Differences in microbial composition between RA patients and healthy individuals may help predict RA development in asymptomatic seropositive individuals. The marked heterogeneity of RF and ACPA associations across cohorts suggests that microbiota–autoantibody relationships are context-dependent and influenced by host, environmental, and methodological factors, limiting their immediate clinical applicability. Currently, RF and ACPA positivity alone cannot reliably identify which patients will progress to clinical RA, resulting in missed opportunities for early intervention. Microbiota-based biomarkers may assist in stratifying patients by their likelihood of responding to specific treatments, improving outcomes while reducing the economic burden on healthcare systems. Another promising avenue is the development of targeted therapies that directly modulate the microbiota. RA patients might one day be classified by their microbial profile and treated using a personalised, precision medicine approach. Although such classifications do not yet exist, techniques like faecal microbiota transplantation and probiotics have already been explored as potential strategies to reverse gut dysbiosis. More research is needed to determine their effectiveness and the optimal stage of disease for intervention. In addition, therapies targeting specific pathways in the microbiota–immune system interaction could reveal new therapeutic potential. Overall, these insights highlight the potential relevance of microbiota-based approaches for future diagnostic and therapeutic strategies in RA.

## ANNEX 1

https://mjrheum.org/assets/files/inpress/MJR-2025-0272_Annex%201.pdf
